# Distance-Based Educational Interventions for Empathy and Compassionate Care: A Systematic Review of International Applications

**DOI:** 10.7759/cureus.93641

**Published:** 2025-10-01

**Authors:** Spyridon Rigatos, Eleni N Albani, Christos D Lionis, Anastasios Tzenalis, Marilena Anastasaki

**Affiliations:** 1 Nursing, University of Patras, Patra, GRC; 2 Nursing, University of Patra, Patra, GRC; 3 Social Medicine, School of Medicine, University of Crete, Greece, Heraklion, GRC

**Keywords:** compassionate care, distance education, e-learning, empathy, healthcare education, mindfulness, virtual reality

## Abstract

Empathy and compassionate care are essential components of high-quality healthcare, yet their integration into clinical education remains inconsistent. With the growing shift toward remote learning, distance-based educational interventions have emerged as promising tools for cultivating these competencies across healthcare professions.

This study systematically reviewed international research examining the design, implementation, and effectiveness of distance-based educational interventions aimed at enhancing empathy and compassionate care among healthcare students and professionals.

A systematic review was conducted in accordance with Preferred Reporting Items for Systematic Reviews and Meta-Analyses (PRISMA) 2020 guidelines. Five databases: PubMed, Scopus, Cumulative Index to Nursing and Allied Health Literature (CINAHL), Web of Science, and Education Resources Information Center (ERIC) were searched for peer-reviewed articles published between January 2015 and June 2025. Eligible studies involved remote educational interventions explicitly targeting empathy and/or compassion with reported outcome measures. Two independent reviewers screened and extracted data on study design, population, intervention type, outcomes, and main findings. A narrative synthesis approach was used.

Twenty-five studies met the inclusion criteria. Intervention types included online mindfulness and self-compassion training (n = 9), virtual reality simulations (n = 6), role-play and simulation-based learning (n = 4), e-learning modules (n = 4), and narrative/reflection-based interventions (n = 1). Study designs encompassed quasi-experimental (n = 8), randomized controlled trials (n = 8), qualitative (n = 3), mixed-methods (n = 4), cross-sectional (n = 1), and retrospective analysis (n = 1). Empathy (n = 11) and self-compassion (n = 7) were the most commonly assessed outcomes. Twenty-one studies reported statistically significant improvements in at least one targeted domain.

Distance-based interventions demonstrate strong potential to foster empathy and compassionate care in healthcare education. When designed to be interactive, experiential, and theory-driven, digital tools can effectively complement traditional curricula. Future research should emphasize methodological rigor, cultural adaptability, and long-term effectiveness to support sustainable integration into health professions education.

## Introduction and background

Empathy and compassionate care are foundational elements in the delivery of high-quality healthcare. Empathy, often defined as the cognitive and affective ability to understand and share the feelings of another, facilitates trust and promotes therapeutic relationships between healthcare providers and patients [1]. Compassion, while closely related, extends beyond empathy to include a motivational component-prompting action to alleviate the suffering of others [2]. These humanistic values are increasingly recognized as critical to improving clinical outcomes, enhancing patient satisfaction, reducing provider burnout, and fostering ethical decision-making in complex care environments [3-5].

Despite their significance, these relational skills remain inconsistently taught in healthcare education, which often emphasizes technical knowledge and clinical proficiency over affective development. Indeed, multiple studies have documented a significant decline in empathy during the course of clinical training, particularly among medical and nursing students [6,7]. Factors contributing to this erosion include academic stress, emotional desensitization, lack of reflective practice, and the overwhelming emphasis on technical competence over interpersonal connection [8,9]. As a result, educational institutions and professional organizations have increasingly emphasized the need to develop and implement targeted interventions aimed at sustaining and enhancing empathic and compassionate competencies throughout healthcare training [10,11].

In response to these challenges, distance-based educational interventions have emerged as a scalable and flexible solution. These interventions utilize online platforms, virtual simulations, and digital tools to deliver structured training in empathy and compassion, either fully remotely or as part of blended learning models. Their relevance has been amplified by the COVID-19 pandemic, which necessitated a global shift toward remote education. Moreover, digital interventions offer unique pedagogical advantages, such as personalized pacing, immersive experiences, and broad accessibility-particularly in under-resourced or geographically dispersed settings. These modalities aim to reproduce complex emotional and clinical encounters in a psychologically safe yet emotionally engaging learning environment [12-14]. Additionally, frameworks such as mindfulness-based training, narrative medicine, and compassion-focused therapy have been successfully adapted to online platforms, offering flexible, scalable solutions for empathy education [15-17].

Nevertheless, the literature in this domain is heterogeneous, spanning diverse populations, pedagogical models, cultural settings, and assessment tools. Systematic synthesis of this evidence is essential to identify effective practices, guide curriculum development, and inform policy-making across health professions education. While several reviews have addressed face-to-face training or simulation-based empathy education, few have focused exclusively on distance-based interventions targeting empathy and compassion-despite their growing relevance and application across international contexts. This systematic review explores the international landscape of such interventions, evaluating their design, implementation, and effectiveness in enhancing empathy and compassionate care among healthcare students and professionals.

## Review

Aim

This systematic review aims to critically examine the international landscape of distance-based educational interventions designed to enhance empathy and compassionate care among healthcare students and professionals. Specifically, it seeks to: (a) identify the types of digital educational approaches employed; (b) evaluate their methodological quality and effectiveness; and (c) provide evidence-based recommendations for the future development and integration of such interventions into health professions education.

Methodology

This study was conducted as a systematic review following the Preferred Reporting Items for Systematic Reviews and Meta-Analyses (PRISMA 2020) guidelines [18].

Studies were eligible for inclusion if they met the following criteria: (a) the study population consisted of undergraduate or postgraduate students in healthcare-related programs or licensed healthcare professionals; (b) the intervention was explicitly designed to enhance empathy and/or compassionate care and was delivered remotely through digitally mediated platforms (e.g., online courses, virtual simulations, immersive technologies); (c) the intervention reported measurable outcomes related to empathy, compassion, or self-compassion; (d) the study was a peer-reviewed journal article published in English between January 2015 and June 2025. Exclusion criteria included studies that lacked empirical data, were not delivered remotely, or focused on unrelated constructs. Opinion papers, protocols, conference abstracts, and grey literature were also excluded.

A comprehensive search was performed in five electronic databases: PubMed, Cumulative Index to Nursing and Allied Health Literature (CINAHL), Scopus, Web of Science, and Education Resources Information Center (ERIC), covering the period January 2015 to June 2025. Additional hand-searching of reference lists and citation tracking were performed for key articles. The following search string was adapted for each database using Boolean operators: (“empathy” OR “compassion” OR “compassionate care”) AND (“education” OR “training” OR “intervention”) AND (“online” OR “distance-based” OR “e-learning” OR “virtual reality” OR “simulation” OR “digital”)

**Figure 1:**
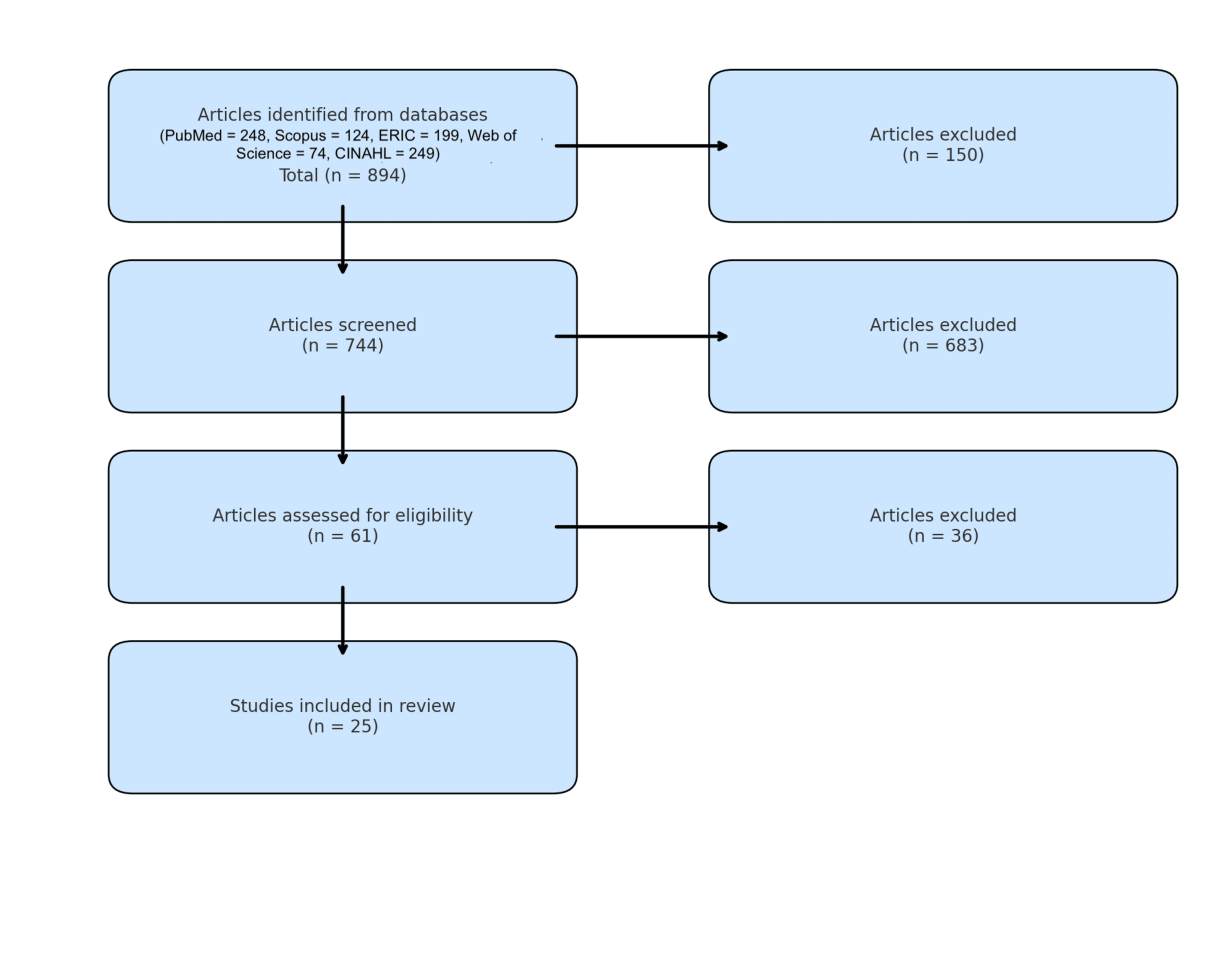
PRISMA 2020 Flow Diagram Illustrating the Study Selection Process The flowchart summarizes the identification, screening, eligibility assessment, and final inclusion of studies in this systematic review, based on PRISMA 2020 guidelines. A total of 894 records were identified through five databases. After removing duplicates (n = 150), 744 articles were screened at the title and abstract level. Of these, 683 were excluded for not meeting inclusion criteria. 61 full-text articles were assessed for eligibility, with 36 excluded for various reasons (e.g., non-empirical design, irrelevant outcomes, or lack of digital delivery). Ultimately, 25 studies met all criteria and were included in the final synthesis.

After removing duplicates, two independent reviewers conducted a blinded screening of all titles and abstracts for relevance. Full-text screening followed independently, with any discrepancies resolved through discussion or adjudication by a third reviewer. The PRISMA flow diagram (Figure 1) illustrates the screening and selection process.

A standardized data extraction form was developed to collect relevant information from each included study: author(s), year of publication, country, study design, participant characteristics, intervention type and duration, outcome measures used, and key findings. One reviewer extracted the data, and a second reviewer cross-checked it for accuracy and consistency.

Given the methodological and conceptual heterogeneity of the included studies, a narrative synthesis was conducted rather than a meta-analysis. Results were grouped thematically based on the type of digital intervention (e.g., virtual reality, online mindfulness, simulation-based training). Where applicable, reported effect sizes and statistical significance were extracted to support the interpretation of intervention effectiveness. As this is a systematic review of published literature, no ethical approval was required. The review was conducted in accordance with the PRISMA 2020 guidelines and adhered to ethical standards for research synthesis, including transparent reporting and appropriate citation of all sources.

Results

The initial database search yielded 894 records, of which 744 remained after removal of duplicates. After title and abstract screening, 683 records were excluded. Full-text assessment was conducted on 61 articles, of which 25 studies met all inclusion criteria and were included in the final synthesis. The reasons for exclusion are detailed in the PRISMA flow diagram (Figure 1).

The 25 studies included in this review employed a variety of methodological designs. A substantial proportion (n = 8) used quasi-experimental or pre-post designs [19-26], often reflecting practical implementation in educational contexts. An equal number (n = 8) utilized randomized controlled trials (RCTs) [17,27-33], which offer a higher standard of internal validity and control. Mixed-methods approaches (n = 4) were also common [34-37], enabling the integration of quantitative outcomes with rich qualitative insights. Qualitative methodologies (n = 3) were primarily used to explore learners’ experiences [38-40], identity development, or the perceived impact of the intervention. One study used a cross-sectional survey to investigate associations between self-compassion and well-being [14], while another adopted a retrospective transcript analysis of virtual simulations to assess empathic communication [41]. The heterogeneity of designs highlights the multifaceted nature of educational research in this domain and supports the need for both rigorous quantitative metrics and reflective qualitative inquiry.

The included studies implemented a diverse range of digital strategies to enhance empathy and compassionate care in healthcare education. Online mindfulness and self-compassion training was the most commonly utilized approach (n = 9) [21-25,28,29,31,33,40], often grounded in evidence-based psychological frameworks such as mindful self-compassion (MSC) or compassion-focused therapy (CFT). Virtual reality (VR) simulations (n = 6) [13,14,24,34,36,38] were also widely adopted, offering immersive, first-person experiences designed to foster perspective-taking and emotional engagement. Role-play and simulation-based learning interventions (n = 4) [27,32,37,41], frequently conducted via remote platforms or using virtual patient avatars, focused on active skill-building and situational practice. E-learning modules (n = 4) [26,30,35,39] provided structured digital content on empathy, ethics, and communication. A small number of studies employed narrative or reflective writing interventions (n = 1) [20], while others adopted multi-component or blended approaches (n = 1) [19] that combined mindfulness, simulation, and storytelling. This diversity in intervention strategies underscores the growing innovation and adaptability of remote compassion training tools, reflecting both technological advancement and pedagogical evolution.

The included studies assessed a diverse range of outcomes related to empathy and compassionate care. Empathy was the most commonly measured construct (n = 11) [13,19,20,24,27,30,34,36-38,41], often using standardized instruments such as the Jefferson scale of physician empathy (JSPE) or its adaptations. A considerable number of studies also evaluated self-compassion and compassion (n = 7) [14,22,25,26,29,33,40], particularly in interventions incorporating mindfulness or compassion-focused approaches. Additionally, reflective capacity and professional identity (n = 4) [20,35,38,39] emerged as key domains, especially in narrative-based or qualitative studies. Measures of stress and anxiety (n = 3) [22,23,33] and knowledge or clinical skills acquisition (n = 3) [24,32,36] were primarily associated with experiential and simulation-based interventions. Fewer studies examined attitudes toward patients or mental illness (n = 2) [24,30], and only one study assessed burnout and compassion fatigue directly [21], and one more assessed acceptability/feasibility [28]. This distribution reflects the multidimensional nature of empathy and compassion, as well as the varied aims of digital educational interventions in healthcare settings.

The majority of the included studies (n = 20) [13,14,19-32,34-37,39] reported statistically significant improvements in at least one targeted outcome related to empathy, self-compassion, or associated psychosocial constructs. These outcomes were typically measured using validated quantitative tools [e.g., Jefferson scale of empathy (JSE), self-compassion scale (SCS), Pro quality of life (QOL)] or supported by qualitative reflections. A smaller subset of studies (n = 3)[33,40,41] documented positive trends that did not reach statistical significance, often due to limited sample sizes or pilot study constraints. One study reported mixed results [38], highlighting both the barriers and the potential positive impact of the intervention. These findings suggest an overall encouraging pattern of effectiveness for distance-based interventions, particularly when multimodal and grounded in experiential learning principles.

**Figure 2:**
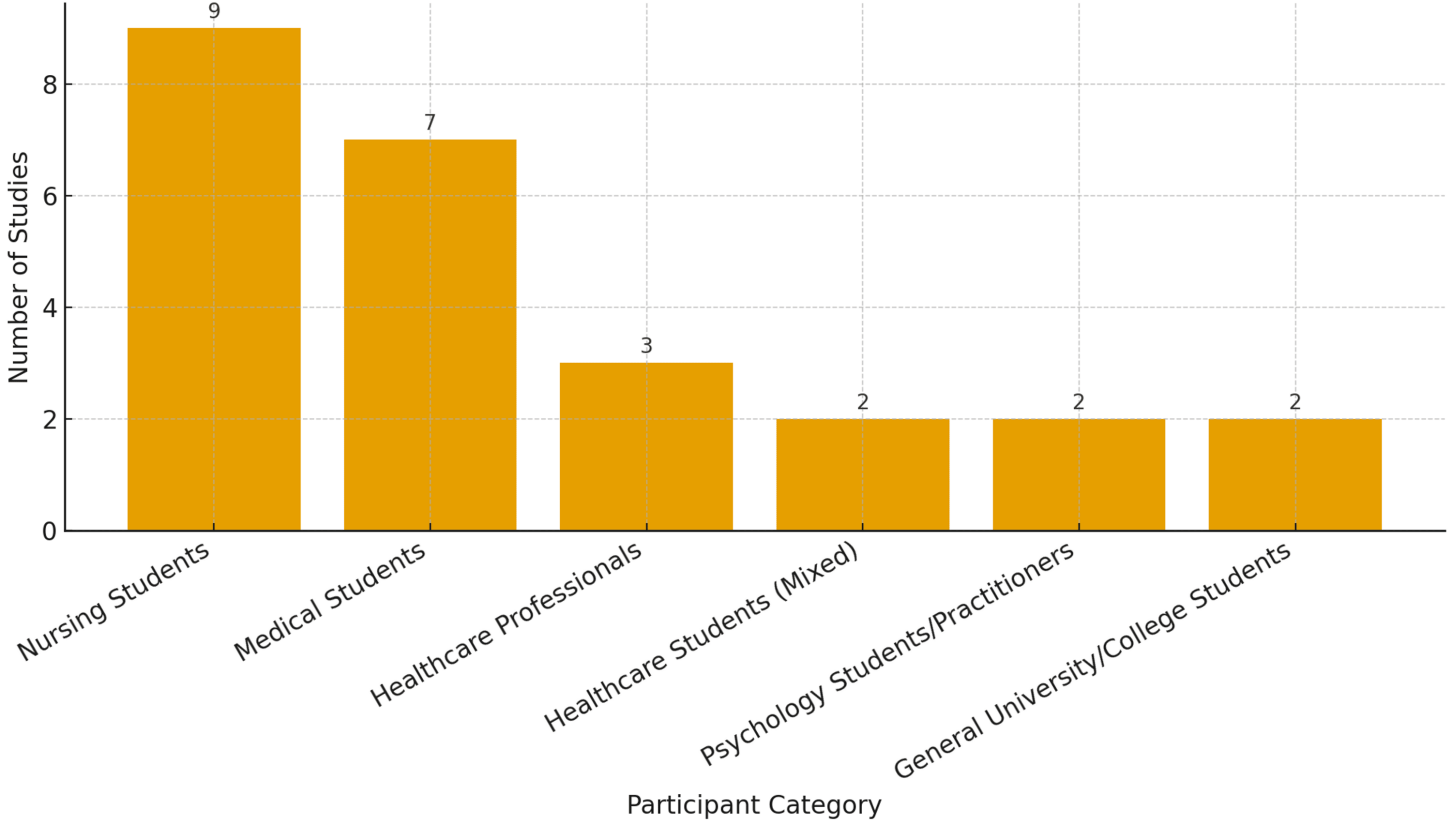
Distribution of Target Populations Across Included Studies (n = 25) Distribution of the 25 included studies based on the primary target population. Nursing students (n = 9) and medical students (n = 7) represented the most frequently studied groups, followed by healthcare professionals (n = 3), healthcare students (mixed) (n = 2), psychology students/ practitioners (n = 2), and general university/college students (n = 2).

The 25 studies included in this review encompassed a diverse range of participant populations across different healthcare education levels and professional roles. The most commonly targeted group was nursing students (n = 9) [14,23,28,33,35,37,38,39,41] followed by medical students (n = 7) [13,19,20,25,27,32,34], reflecting the central role of these two disciplines in the development of empathy and compassionate care competencies. Several studies included mixed groups of healthcare students (n = 2) [24,36], allowing for interdisciplinary engagement. A smaller number of interventions targeted practicing professionals (n = 3) [21,30,40], such as oncology nurses and psychologists, as well as psychology students or practitioners (n = 2) [22,29], and general university students (n = 2) [26,31]. This distribution suggests that while the majority of interventions are focused on early-stage professional education, there is growing interest in extending compassion training across different stages of healthcare training and practice (Figure 2).

**Figure 3:**
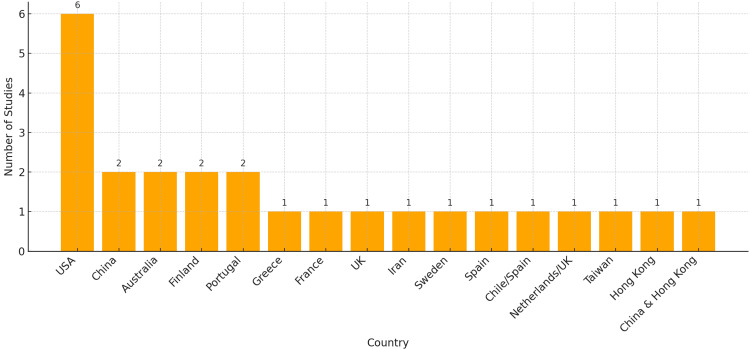
Country of Origin of Included Studies (n = 25) This figure illustrates the geographic distribution of the 25 studies included in this systematic review. The country of origin was determined based on the authors’ institutional affiliations or information provided in the article. In cases where multiple affiliations were reported, each country was counted. The majority of studies originated from the United States (n = 6), followed by China (n = 2), Australia (n = 2), Finland (n = 2), Portugal (n = 2), and several single-country contributions (e.g., Greece, Iran, UK, Sweden, Taiwan, etc.). The diversity of countries underscores the global interest in using digital tools to foster empathy and compassionate care in healthcare education.

The included studies demonstrated substantial geographic diversity, reflecting a growing global emphasis on remote compassion education. The United States led in frequency, contributing six studies, followed by multiple countries with one or two studies each, including China, Australia, Finland, Portugal, and others. Several studies reflected cross-national collaborations, highlighting shared pedagogical interests. The findings suggest that distance-based interventions for empathy and compassion are not limited to any single cultural or healthcare context, enhancing the potential for international adoption and scalability (Figure 3).

The characteristics of the included studies are summarized in Table 1. The 25 studies encompassed a broad range of geographic regions, participant groups (including medical students, nursing students, healthcare professionals, and psychology students), and intervention types. Study designs varied from randomized controlled trials and quasi-experimental designs to qualitative and mixed-methods approaches. Interventions were implemented through digital platforms and delivered remotely, with most targeting improvements in empathy, self-compassion, or related psychosocial competencies. The diversity of both methods and contexts illustrates the growing international interest in integrating digitally mediated compassion training into health professions education.

**Table 1:** Study Characteristics of Included Articles JSE: Jefferson scale of empathy; RCT: randomized controlled trial; MSC: mindful self-compassion (program); STAR: skills training and reskilling;  VR: Virtual reality; ANOVA: analysis of variance; MB-CARE: mindfulness-based care; ESGC: eight steps to great compassion; BLS: basic life support; JSPE: Jefferson scale of physician empathy; SCS: self-compassion scale; QOL: quality of life FFMQ: five facets of mindfulness questionnaire; PSS: perceived stress scale; SMBQ:shirom-melamed burnout questionnaire; CPTSD: complex posttraumatic stress disorder; DSO: disturbances in self-organization; CSES: comprehensive state empathy scale; UX: user experience; IAT: implicit association test; TMS: Toronto mindfulness scale.

Study (Author, Year)	Country	Study Design	Population	Intervention/Technology	Outcomes Measured	Key Findings
Alieldin et al. [34]	United States of America (inferred from affiliation)	Mixed methods, pre–post (JSE), with 6‑month follow-up, thematic interviews	1st-year medical students (n = 19)	Immersive VR empathy training scenario—social isolation of older adult; includes debriefing session	Empathy via JSE (quantitative); participant experiences via semi‑structured interviews (immediate and 6‑month qualitative follow-up)	Significant increase in empathy (Δ = +5.94, p < 0.01); strong acceptance of IVR as a teaching tool; immersion, presence, embodiment cited as key mechanisms enhancing empathy; long-term value retained at 6‑month follow‑up
Avlogiari et al. [19]	Greece	Pre‑post experiential training design (no control)	Medical students, n = 47 (Greek medical students, 4th year and above)	“Empathy in Healthcare”—intensive 20‑hour experiential training (30% lectures, 70% role-play, empathy game, based on mediation techniques)	Empathy via JSE‑S (Greek version); baseline, post‑training, and 6‑month follow-up	JSE‑S increase of 11.3 points post‑training (p
Brun et al. [40]	United States of America (inferred from affiliation)	Qualitative study (10 post-training interviews)	Healthcare professionals (MB-CARE program participants)	Mindfulness- and compassion-focused training (MB‑CARE)	Self- and patient-compassion, equanimity, reactivity, caring listening	Training enhanced compassion, attention, calmness, and reduced emotional reactivity
Buffel du Vaure et al. [27]	France	RCT (in-person role‑play)	Medical students	Role-play empathy training	Empathy (JSPE)	Significant increase (p = 0.04)
Coster et al. [28]	United Kingdom	Pilot RCT feasibility	Nursing students (n=77)	Online mindfulness (5 modules)	Completion rates, acceptability	Low completion; positive feedback on stress, focus
Daryazadeh et al. [20]	Iran	Quasi-experimental pre-post	Medical students	Narrative medicine program	Reflection (REFLECT), Empathy (JSPE)	Significant improvements (p < 0.001)
Duarte et al. [21]	Portugal	Non-randomized pre-post	Oncology nurses	Mindfulness-based intervention	Burnout (MBI), Compassion fatigue (ProQOL)	Significant reductions in both
Eriksson et al. [29]	Sweden	RCT (web-based MSC)	Practicing psychologists (n = 101)	6‑week online MSC	SCS, FFMQ, PSS, SMBQ	Large effects: self-compassion d = 0.86–0.94; moderate: mindfulness, stress, burnout
González‑García [22]	Spain	Pre-post feasibility	Psychology students (n = 66)	16-day online mindfulness-compassion	SCS, stress, anxiety	Improved self-compassion, reduced stress & anxiety (p < 0.001)
Gutierrez‑Carmona [23]	Chile/Spain	Pre-experimental	Nursing students (n ≈ 45)	Compassion & mindfulness training	Stress, state/trait anxiety	Stress & state anxiety ↓, trait anxiety ↓; self‑compassion mediates effects
Hattink et al. [30]	Netherlands & United Kingdom	RCT e-learning (STAR)	Dementia caregivers	Web-based STAR portal	Empathy domains, caregiver attitudes	Improved empathy and person-centered care attitudes
Hofmeyer et al. [35]	Australia	Mixed methods evaluation (quasi-experimental + qualitative feedback)	undergraduate nursing students (n = 29)	Online compassion literacy module embedded in 13-week ethics course	Qualitative reflections, professional identity, and understanding of compassion	The intervention enhanced students’ understanding of compassion and ethics, supported reflective practice, and contributed to their professional identity development.
Hofmeyer et al. [39]	Australia	Qualitative	Nursing students (n = 62)	Online compassionate care course	Reflective journals	Heightened awareness of compassionate care
Huang et al. [31]	China	Pilot RCT online	College students (n = 77)	10-day brief self-compassion intervention	CPTSD total, DSO symptoms	Significant CPTSD & DSO reduction; feasible intervention
Koivisto et al. [14]	Finland	Cross-sectional	Nursing students	Immersive VR simulation game	CSES, UX metrics	Positive empathy and user experience outcomes
Lin et al. [13]	Taiwan	Randomized case–control	Medical students (~59)	VR depressed-student simulation	JSE domains, IAT, Immersion metrics	Perspective-taking & compassionate care ↑; patient shoes ↓; immersion correlated
Liu et al. [36]	Hong Kong	Multiple-methods	Healthcare students	VR simulation (older adults)	Empathy, knowledge gain	Significant empathy & knowledge improvement
Marques et al. [24]	Portugal	Quasi‑experimental pre‑post with control (VR vs. 2D video)	Health students (n = 102)	Immersive VR simulation reproducing psychotic symptoms vs. 2D video	Empathy, attitude toward schizophrenia, mental health knowledge	Both VR and video improved outcomes, but VR produced greater positive changes in attitudes and knowledge
Mattsson et al. [38]	Finland	Qualitative	Nursing students (n = 20)	VR simulation game	Qualitative empathy reflections	Empathy boosted via realism and reflection; tech-focus barrier
Mehta et al. [25]	United States of America	Comparative pre-post	Medical students (4th year)	8-session mindfulness-compassion toolkit	SCS, Compassion Scale, TMS	Significant gains in self-compassion (+8.7), compassion (+6.0), mindfulness (+4.4; all p < 0.05)
Ochs et al. [37]	United States of America	Mixed‑model ANOVA pre‑post (role‑play + group activities vs control)	Nursing students in a community/population health course	Role‑play simulation and group interactivities simulating care for people with disabilities	Empathy (Revised Kiersma‑Chen Empathy Scale)	Significant improvement in empathy scores post‑intervention in the experimental group
Strekalova et al. [41]	United States of America	Retrospective transcript analysis	Nursing students (n = 343)	Virtual patient simulation	Empathic responses	Only 33.5% recognized as empathic; “conventional” most frequent
Tendhar et al. [26]	United States of America	Single-group pre‑ and post‑test quantitative design	Undergraduate university students (n = 92; mean age ≈ 20.4)	ESGC – online video-mediated compassion training program	Self-compassion, compassion for others, well-being (PERMA-Profiler), loneliness, physical health, negative emotions	Significant increases in self-compassion, compassion for others, and well-being; decreases in negative emotions and loneliness; 88% reported positive life changes post-program
Wei et al. [32]	China	RCT	Undergraduate medical students (n = 527; control = 264, virtual simulation = 263)	Virtual simulation teaching for BLS grounded in Kolb’s Experiential Learning Model	Practical skill performance (objective exam scores), self-assessment, course satisfaction (Likert scale), effect size (Cohen’s d = 0.29)	The virtual simulation group significantly outperformed the traditional teaching group in practical skills (p < 0.01, d = 0.29), self-evaluation (p < 0.01), and course satisfaction (high levels across multiple dimensions).
Yang et al. [33]	China & Hong Kong	Pilot RCT online	Nursing students (n = 28)	8-week WeChat self-compassion training	Mindfulness, self-compassion, stress	Mindfulness ↑; positive trends in SCS/stress; high engagement

Discussion

This systematic review synthesizes the findings of 25 international studies evaluating distance-based educational interventions designed to enhance empathy and compassionate care among healthcare students and professionals. The evidence demonstrates that such interventions are not only feasible but also generally effective in cultivating core affective and relational competencies that are often underemphasized in traditional healthcare curricula.

A key finding of this review is the broad diversity of digital pedagogical strategies employed across the included studies. Interventions ranged from immersive VR, which promoted perspective-taking and emotional immersion, and web-based mindfulness programs, which improved stress management, emotional regulation, and compassionate responses, to narrative-based learning, online role-play, and reflective exercises that helped learners process ethical complexity and patient suffering in a psychologically safe format. Multimodal approaches-particularly those integrating experiential, reflective, and mindfulness-based components-appeared especially effective in promoting empathic understanding and compassionate behavior.

In terms of measured outcomes, the most frequently assessed domains were empathy (e.g., via JSE), self-compassion (e.g., SCS, MSC), and psychological well-being (e.g., stress, anxiety, burnout). The majority of studies reported statistically significant improvements in these domains post-intervention. Notably, interventions that combined mindfulness training with structured reflection or digital simulation consistently yielded moderate to large effects, suggesting that emotion-regulation mechanisms may mediate the development of compassionate care. This aligns with pedagogical research suggesting that empathy and compassion are not simply innate traits but can be fostered through intentional, experiential learning environments. However, the validity and sensitivity of certain measurement tools in digital contexts warrant further scrutiny. Instruments such as the JSPE and SCS, while widely used, were originally developed for face-to-face clinical training and may not fully capture the nuances of remote experiential learning. Future studies should critically appraise whether these tools adequately reflect changes in empathy and compassion elicited by digital methods, or if adaptations and new metrics are needed for online environments.

The thematic analysis of intervention strategies revealed several effective clusters, including immersive simulations (especially VR), online mindfulness and self-compassion programs, and digital storytelling platforms. These approaches not only supported cognitive perspective-taking but also fostered emotional resonance and embodied understanding, particularly when learners were placed in the virtual “shoes” of patients or vulnerable individuals. The results of this review complement and extend the findings of prior reviews that focused on face-to-face or simulation-based empathy training. For example, Levett-Jones et al. and Patel et al. confirmed the effectiveness of empathy curricula in nursing and medical education but did not isolate distance-based modalities [42-43]. In contrast, this review demonstrates that digital interventions are not merely substitutes, but in some cases, they offer unique pedagogical affordances-such as scalability, standardization, and personalized pacing. Notably, recent work by Sinclair et al. (2021) also underscored the need for compassion education to be longitudinal, culturally contextualized, and integrated across the curriculum, observations that resonate with the findings here [44].

Moreover, the relevance of distance-based empathy and compassion training has been heightened in the wake of the COVID-19 pandemic. Healthcare students and professionals have faced unprecedented emotional strain, reduced clinical exposure, and increased reliance on remote learning. These realities make the case for integrating scalable, effective learning interventions into health professions education more urgent than ever. Digital formats can fill critical gaps in relational skill development when in-person interactions are limited, while also offering avenues for self-paced, reflective learning tailored to individual needs and emotional readiness.

Importantly, this review highlights the potential of digital tools to democratize access to compassion training, especially in settings where in-person training may be logistically or financially constrained. The flexibility and scalability of online interventions allow for integration into blended learning curricula and continuing professional development programs. However, digital equity and learner engagement remain key considerations in program implementation. More comparative studies are needed to explore how different digital formats influence learning outcomes and whether certain strategies are more effective for particular learner populations, professional roles, or cultural settings.

Limitations

Despite its strengths, this review is subject to several limitations: Heterogeneity in outcome measures and intervention types limited meta-analytic synthesis; Several studies lacked control groups or long-term follow-up, restricting inferences about sustained impact; Self-report instruments were frequently used, raising concerns about social desirability and measurement bias; Although global in scope, certain regions (e.g., Africa, Southeast Asia) were underrepresented; This review offers a broad overview rather than an in-depth analysis. It does not assess the risk of bias due to the methodological variability of the included studies. Future reviews may benefit from broader language inclusion, standardized outcome metrics, and incorporation of gray literature.

Implications for practice and research

For educators, this review supports the integration of digital compassion and empathy curricula as a core component of professional training, especially in contexts where in-person instruction is limited. Modular, theory-based interventions can be embedded within existing online platforms to enhance relational competencies without sacrificing flexibility.

For researchers, there is a need for: More rigorous trials with longer-term outcomes and cross-cultural validation; Development of blended models combining online learning with supervised clinical practice; Enhanced tools to measure behavioral expressions of empathy and compassion, not just cognitive or emotional self-reports; Evaluation of cost-effectiveness and scalability of digital interventions, especially in low-resource settings.

## Conclusions

This systematic review highlights the growing potential of distance-based educational interventions to effectively cultivate empathy and compassionate care in healthcare education. The findings underscore the versatility and adaptability of digital approaches-such as immersive simulations, online mindfulness programs, and narrative-based modules-in promoting affective, interpersonal, and reflective competencies across diverse healthcare learner populations.

Despite heterogeneity in design and outcomes, the majority of included studies reported significant improvements in empathy, self-compassion, or related psychological domains. Interventions that were interactive, experiential, and grounded in established theoretical frameworks appeared particularly impactful. Moreover, the use of digital tools offers scalable, accessible, and cost-effective avenues for integrating compassion training into both undergraduate and continuing professional development curricula, especially in remote or resource-limited settings.

The COVID-19 pandemic has fundamentally reshaped the landscape of healthcare education, accelerating the adoption of online learning while simultaneously intensifying emotional burdens on both students and professionals. In this context, distance-based interventions are not merely an alternative-they are a timely and necessary response to contemporary challenges such as increased psychological distress, disrupted clinical exposure, and the need for emotional resilience. Embedding compassion-focused education within digital modalities offers a pragmatic pathway to support the mental well-being and relational competence of future and current healthcare providers.

However, the field would benefit from more methodologically rigorous research, including randomized controlled trials, standardized outcome measures, and long-term follow-ups to assess the sustainability of training effects. Additionally, future studies should explore cultural and contextual factors that influence learners’ responsiveness to digital interventions and consider co-design approaches to enhance learner engagement and relevance.

In sum, distance-based compassion training represents a promising frontier in health professions education. When implemented thoughtfully and evaluated rigorously, these interventions can contribute meaningfully to the development of empathic and humanistic healthcare providers prepared to meet the relational demands of modern clinical practice. In a time when both patients and providers face increasing emotional strain and systemic challenges, integrating technology and humanity in healthcare education is not only possible-it is imperative.
